# Evaluation of the Executive Functioning and Psychological Adjustment of Child-to-Parent Offenders: Epidemiology and Quantification of Harm

**DOI:** 10.3389/fpsyg.2021.616855

**Published:** 2021-04-09

**Authors:** Ricardo Fandiño, Juan Basanta, Jéssica Sanmarco, Ramón Arce, Francisca Fariña

**Affiliations:** ^1^Departamento Análisis e Intervención Psicosocioeducativa (AIPSE), Universidad de Vigo, Vigo, Spain; ^2^Psicología Organizacional, Jurídica Forense y Metodología de las Ciencias del Comportamiento, Universidad de Santiago de Compostela, Santiago de Compostela, Spain; ^3^Facultad de Psicología, Universidad de Santiago de Compostela, Santiago de Compostela, Spain

**Keywords:** MMPI-A, stroop tasks, juvenile offender, prevalence, child-to-parent violence

## Abstract

With the aim of ascertaining if child-to-parent offenders have impairments in the executive functions and psychological maladjustment, and to quantify the potential harm and epidemiology, a field study was designed. As for this, 76 juvenile offenders sentenced for child-to-parent violence were assessed in executive functions (Stroop tasks) and psychological adjustment (Minnesota Multiphasic Personality Inventory-Adolescent, MMPI-A). The results showed valid responses for 75 juveniles and that data were not generally biased in line with defensiveness or malingering (differential diagnosis in justice juvenile evaluations). In psychological adjustment, the results revealed a significantly higher maladjustment among offenders on all the basic clinical scales with 23% more symptoms of hysteria than the normative population, 37% more of depressive symptoms, 44% more of hypochondriac symptoms, 68% more of psychopathic deviation symptoms, 46% more of paranoid symptoms, 26% more of psychasthenic symptoms, 24% more symptoms of schizophrenia, 17% more symptoms of hypomania, and 13% more symptoms of social introversion. Epidemiologically, the prevalence rates of clinical deterioration were significantly greater than expected (0.05 in normative sample) in hypochondria (28.0%), depression (29.3%), hysteria (29.3%), psychopathic deviation (60%), paranoia (30.7%), psychasthenia (22.7%), and schizophrenia (25.3%). As for the cognitive functions, the offenders exhibited impairments estimated at 62.0% in word reading, 47.9% in color naming, 45.8% in color-word, and 11.9% in interference and a significantly higher prevalence of caseness than expected in word reading (65%), color naming (71%), and color-word (70.2%). The implications of the results for intervention are discussed.

## Introduction

Over 60 years after Sears et al. (1957) drew attention to child-to-parent violence (CPV), there is a wealth of literature on domestic violence, particularly intimate partner violence, but research on CPV is paradoxically scarce. CPV is defined as violence exerted by a child on a parent, whereby a child is defined as person under the legal adult age of 18 years (it may be extended to < 21 years for reoffenders). This excludes CPV committed by offspring over 18, the vast majority of whom continue to live with parents. The literature has mainly focused in the predictors of CPV (e.g., age and gender of the perpetrator, parenting style, type of violence, exposure to family violence, parent-to-child violence, and socio-economic status) and the sociodemographic characteristics (e.g., age and gender) of the child-to-parent offenders (CPOs) (Gallego et al., 2019; Hoyo-Bilbao et al., 2020; Perez-Gramaje et al., 2020). The results have provided the identification of the risk factors of CPV, which are variables predicting the high probability of child-to-parent violent behavior, and to a much lesser extent risk-based protective factors, which are variables predicting a low probability of CPV among a risk group, or interactive protective factors, i.e., variables that nullifies the effect of the risk factors (Gallego et al., 2019; Cortina and Martín, 2020; Loinaz and Sousa, 2020; Loinaz et al., 2020). Both risk and protective factors are classified as either dynamic factors that are susceptible to intervention (e.g., parenting style and substance abuse) or static factors that are not susceptible to intervention (e.g., previous parent-to-child violence and gender). In offender intervention programs, dynamic factors are considered to be needs that must be the target of interventions. Cognitive behavioral intervention programs have proven to be the most effective in the intervention of juvenile and adult delinquency (Koehler et al., 2013; Arce et al., 2020), often targeting psychological maladjustment and cognitive competence as these needs are significant predictors of aggression, delinquency, and recidivism in delinquency (Wibbelink et al., 2017; Basanta et al., 2018; Perez-Gramaje et al., 2020). Remarkably, the contents of treatments for CPOs have been barely assessed, even though CPO interventions should be the primary objective according to judicial sentences. Of the array of needs identified in the literature on adult and juvenile violence and delinquency, psychological adjustment (Mayorga et al., 2020; Beaudry et al., 2021) and cognitive competency (Arias et al., 2020; Beelmann and Lösel, 2020) in CPOs have received little attention, and the results on CPV are inconsistent (Simmons et al., 2018).

Meta-analyses have found violent and antisocial psychopaths have deficits in neuronal activity and abnormal activity in the pre-frontal cortex (Yang and Raine, 2009). Moreover, impaired executive functioning is a predictor of recidivism in delinquency and, by extension, of life persistence (Miura and Fuchigami, 2017), and the magnitude of the deficit in executive functioning varies according to the type of antisocial behavior, i.e., an effect size ranging from *d* = 0.94 for criminality, 0.78 for delinquency, 0.36 for conduct disorder (CD) to 0.25 for psychopathy (Morgan and Lilienfeld, 2000). A more recent and broader meta-analysis that reported lower effect sizes for criminality, *d* = 0.61, and for delinquency, *d* = 0.41, meanwhile informed higher effect sizes for CD, *d* = 0.54, and for psychopathy, *d* = 0.42 (Ogilvie et al., 2011). On the basis of these results, the deficits in executive functioning can be quantified as 42.5% for criminality, 36.3% for delinquency, 17.7% for CD, and 12.4% for psychopathy in the meta-analysis of Morgan and Lilienfeld and 29.2% for criminality, 20.1% for delinquency, 26.1% for CD, and 20.6% for psychopathy in the meta-analysis of Ogilvie et al. These results were significantly different [95% confidence intervals (CIs) for the means did not overlap]; however, impairment in executive functions (EFs) was significant in all types of antisocial behavior (ASB) in both meta-analyses. The measure of EFs is a controversial issue since they are understood as either a series of cognitive processes and behavioral competences that regulate the execution of complex tasks or an anatomical concept that locates executive functioning in the frontal lobe. Though both concepts are intrinsically related, impaired executive functioning is associated with other areas of the brain, whereas the optimum executive functioning involves the entire brain (Collette et al., 2005). Regardless of the perspective, the most relevant from a practical point of view is not so much the anatomical location of injury but impairments in EFs. Though the measure of cognitive processes and skills requires a conceptual model widely accepted by the scientific community, it has not been developed owing to the lack of consensus regarding the cognitive processes and skills involved in EFs (Jurado and Rosselli, 2007) and the absence of a reliable measurement instrument (Ogilvie et al., 2011). On the ground of these limitations, Morgan and Lilienfeld (2000) reviewed the well-validated tests for measuring EF impairment (i.e., the category test of the Halstead-Reitan Neuropsychological Battery, the qualitative score on the Porteus Maze Test, the Stroop Interference Test, Part B of the Trail Making Test, the perseverative error score on the Wisconsin Card Sorting Test, and Verbal Fluency Tests) and found the highest sensitivity for the Stroop and Mazes tests.

Bearing this context in mind, a field study was carried out on juveniles convicted of CPV in order to ascertain if the deficits in executive functioning observed in individuals exhibiting antisocial behavior were also observable in CPOs and to quantify the potential harm and epidemiology. Moreover, the psychological adjustment of this population of juveniles was assessed, the harm to mental health quantified, and the clinical epidemiology examined.

## Methods

### Participants

A total of 76 correctional juveniles convicted of CPV, 51 males (67.1%) and 25 females with an age range of 14–20 years (*M* = 16.33, SD = 1.10), participated in this study. In terms of sentencing, three juveniles were serving custodial sentences, seven were in a Secure Children's Home, 61 on Youth Rehabilitation Orders, and five on probation. Of the 76 convicted juveniles, 23 were CPV reoffenders.

### Procedure and Design

The data were gathered in Galicia (northwest of Spain) from court files and from the Young Offender Institutions (YOIs) and the Youth Offending Teams (YOTs) during judicial proceedings or the reception stage in the YOI. The data were obtained with written informed consent of the courts, YOIs, and YOTs and were anonymized. The data was stored and processed in accordance with the Spanish Data Protection Law. Only cases where the conviction was exclusively related to CPV were included. The design sensitivity analysis for the comparison of means with a one-sample *t*-test of a sample of 75 subjects for a medium effect size (*d* = 0.5) showed the probability of detecting (1 – β) significant differences (α < 0.05) was 99.6%. The design sensitivity for the contrast of cases with a constant (clinical deterioration, and moderate and clinical deterioration) showed that, for a medium effect size [odds ratio *(OR)* = 2.47] and a sample of 75 subjects, the probability of obtaining a significant rate (one tailed: higher prevalence among CPOs) was 64.4% for a constant of 0.05 and 85.0% for a constant of 0.10.

### Measurement Instruments

Psychological adjustment was evaluated using the Minnesota Multiphasic Personality Inventory version for adolescents, the MMPI-A, which is the instrument of reference in forensic evaluation and for judicial samples. This instrument not only measures psychological adjustment on nine (masculinity-femininity scale was omitted as it is not a measure of psychological adjustment; Graham, 2011) basic clinical scales [i.e., hypochondriasis (Hs), depression (D), hysteria (Hy), psychopathic deviate (Pd), paranoia (Pa), psychasthenia (Pt), schizophrenia (Sc), hypomania (Ma), and social introversion (Si)] but also the consistency [i.e., True Response Inconsistency (TRIN) and Variable Response Inconsistency (VRIN) scales and |*F1*–*F2*| index] and accuracy [i.e., the self-unfavorable reporting of psychopathology scales (*F, F1, F2*, and *K* scales and the *F*-*K* index) and the self-favorable reporting of psychopathology (i.e., *L* and *K* scales and the *F*-*K* index)] of item responses (Greene, 2011). Indeed, in this type of population, malingering (American Psychiatric Association, 2013), defensiveness (Fariña et al., 2014), and a combination of both should always be suspected (Osuna et al., 2015). The Spanish adaptation and norms of the MMPI-A were employed (Butcher et al., 2003).

The Spanish adaptation of the Stroop Color-Word Test (Golden, 2005) was used to assess executive functioning (cognitive impairments). It consists of three tasks that subjects have to read as fast as possible in 45 s: two congruent and one incongruent. In the congruent conditions, subjects are required to read the names of colors printed in black ink (word reading) and to name the presented colors (color naming). In the incongruent task (color-word), words are printed in an inconsistent color ink, and participants are required to name the color of the ink. The accounts of the read words (W), colors (C), and color-words (CW) are the raw scores. The predicted color-word interference score is calculated from

$$\begin{array}{l} \text{C}{\text{W}}^{\prime }=\left(\text{C }\times \text{ W}\right)/\left(\text{C }+\text{ W}\right) \end{array}$$

The difference between the account of color-word score and predicted color-word score is the interference score, IS = *CW* – *CW*′.

### Data Analysis

The means of the CPOs in the psychological dimensions and the Stroop tasks were compared with test values (one-sample *t*-test) taken from the normative population (Spanish norms) and the means of justice juvenile samples. The normality of the distributions of the variables was verified with asymmetry and kurtosis (<-2 and 2; George and Mallery, 2010). In this study, the justice juvenile samples and the normative population were preferred to a control group as they were less biased than a control group (Schmidt and Hunter, 2015; Novo et al., 2019). Moreover, the study of cases should be carried out with the normative population. Although many comparisons were computed, multiple corrections test was not performed as the grouping factor had one or two levels. Effect sizes were calculated in Cohen's *d* and were interpreted in terms of the probability of superiority of the effect size (PS_ES_; Monteiro et al., 2018). The quantity of harm on the clinical dimensions was calculated by interpreting the effects in the binomial effect size display (BESD; Rosenthal and Rubin, 1982), using *r* (Corrás et al., 2017). The probability of CPOs having more symptoms than the normative population was estimated by the area under the curve (AUC), whereas the probability of asymptomatic CPOs was determined by transforming the effect size to *Z* score and then estimating the probability of an inferiority score (PIS), i.e., estimating the probability of the CPO sample (normal distribution; non-significant K-S for the clinical scales) obtaining a score below the mean of the normative population (normal distribution), PIS = 1 – [NORMSDIST(*Z*)]. For this proportion, the confidence interval (CI) was obtained, and if the CI comprises zero, the rate of asymptomatic cases was zero; if the CI comprises 0.05, it was not significant (trivial); and if the lower limit of the CI was above 0.05, it was significant.

The case study was analyzed (*Z* scores) contrasting the observed probability with a test value: 0.05 for clinical deterioration and suspected malingering (corresponding to a *T* score of ≥ 66.45 or ≤ 33.55) and 0.10 for clinical and moderate deterioration (corresponding to a *T* score of 62.8). Effect sizes were estimated in odds ratio, and the magnitude was interpreted in terms of epidemiology with the effect incremental index (EII; Redondo et al., 2019), (*p*_1_ – *p*_2_)/*p*_1_ where *p*_1_ is the observed probability of caseness in the CPO sample and *p*_2_ is the probability of caseness in the normative population (test value, 0.05 or 0.10). The result of the equation multiplied by 100 was the percentage increase of caseness among the sample of CPOs above the normative sample.

As for *F*-*K* index, norms were not available. Thus, *F* and *K* raw scores were standardized in *Z* scores with the Spanish norms, and the AUC of each score was computed. If the sum of both AUCs was over 0.90, it meant that 90% of the total scores were under the curve, the remaining 10% being out of normality. This cut-off score (±0.90, depending on the sign of the difference between *F* and *K*) was the test value for mean comparisons and the criterion for defensiveness (negative difference) or malingering (positive difference). Likewise, the reliability of this index has not been reported in the literature. Thus, the reliability of the composite of *F* and *K* was computed with the formulas from Mosier (1943).

## Results

### Invalid Protocols

The protocols of the MMPI-A were scrutinized to determine if they had been subjected to extreme acquiescence (TRIN *T* ≥ 80), random responses (VRIN *T* ≥ 80; *F, F1*, or *F2 T* ≥ 120), lack of collaboration (>10 did not respond or double response items) or outliers [*L* raw score (rs) > 12 or *K* rs > 29, i.e., percentile 99.9], in order to eliminate these from the study (Graham, 2011; Greene, 2011; Arce et al., 2015). A case was excluded from the study as the VRIN and TRIN were over a *T* score of 80 (random responses). Likewise, the Stroop tasks were reviewed with the aim of detecting lack of cooperation (inability to read; incomplete tasks) or invalid protocols (raw color-word scores higher than raw word reading or color naming scores). One protocol was identified as invalid and eliminated (the same as in MMPI-A).

### Item Response Consistency

TRIN and VRIN validated all the protocols (rs < 14), and the sample of CPOs showed a consistent response pattern (|*F1*–*F2*|) throughout the test and the measure (test value = 16.45, i.e., in *T* scores, 50 ± 16.45 comprises 90% of the distribution, ruling out 5% in each tail), *t*(74) = −7.06, *p* < 0.001, *d* = 0.82. Nevertheless, not all of the offenders maintained this response pattern throughout the test. In fact, the probability of finding scores above 16.45 in the normative population was 10%, while for CPOs, it was 20% (*n* = 15), with the observed probability being higher than expected for the normative population, *Z*(*N* = 75) = 2.89, *p* < 0.01, OR = 2.0.

### Item Response Accuracy

First, the reliability of the *F-K* index (reliability of the weighted composite) was calculated to determine both the reliability and the estimated error. The results showed a good reliability of 0.788, accounting for the 62.1% of the variance.

The scales and indexes for the measurement of defensiveness, *L* and *K* scales and the *F-K* index (see Table 1), reported that the population of CPOs did not have biased responses in line with defensiveness (negative significant *t*-scores with a cut-off score for defensiveness, i.e., *T* = 66.45 for the scales and rs = −22.48 for the *F-K* index). Likewise, the *F, F1*, and *F2* scales and the *F-K* index for the assessment of malingering showed CPOs did not have biased responses in line with malingering. Nevertheless, the case study (see Table 1) found a significantly high rate of cases (difference between the observed proportion of suspect of malingering or defensiveness and the predicted proportion in normative sample with the cut-off scores, 0.05) in the *L* (defensiveness) and *F, F1*, and *F2* (malingering or severe psychopathology) scales. However, the rate of cases was significantly higher in *F1* than in *F2*, χ^2^(1, *N* = 75) = 18.38, *p* < 0.001. Thus, the classification of malingering or severe clinical cases was mainly associated with the basic clinical scales (*F1*) as compared to the complementary and content scales (*F2*). Moreover, low scores on the *K* scale were related to malingering. However, this criterion on the MMPI-A has not been assessed, so there is no classification cut-off score. Thus, the statistical criterion for the classification of abnormality (*T* ≤ 33.55 resulting from 50 to 16.45, which classified 5% of the normative distribution below this level) was used to quantify the rate of cases in CPOs (*n* = 6, 8%), which was not significant, *Z*(*N* = 75) = 1.19, *ns*.

**Table 1:** One-sample *t*-test for the comparison of the scales and indexes of malingering and defensiveness with a test value (cut-off score) and the contrast of the observed probability of malingering or defensiveness classification among CPOs with a constant (0.05).

**Scale/index**	**Cut-off score**	***t***	***M***	***d***	***f*(*p*)**	***Z***	***OR***
*L*	≥66.45	−9.86^***^	53.55	−1.14	9(0.120)	2.78^**^	2.40
*F*	≥66.45	−5.78^***^	58.95	−0.67	19(0.253)	8.06^***^	5.06
*K*	≥66.45	−12.55^***^	49.44	−1.45	5(0.067)	0.68	1.34
*K*	≤33.55	11.72^***^	49.44	1.37	6(0.080)	1.19	1.60
*F1*	≥66.45	−2.47^*^	62.93	−0.28	32(0.413)	14.42^***^	8.26
*F2*	≥66.45	−10.25^***^	54.63	−1.18	9(0.120)	2.78^**^	2.40
*F-K^+^*	≥0.90	−10.84^***^	0.22	−2.64	5(0.067)	0.68	1.34
*F-K*	≤-0.90	17.77^***^	0.22	3.45	0(0.000)	–	–

*df(74). M, mean of the CPO group; d, Cohen's d; Z, zeta for the difference between the observed proportion and a constant (0.05); OR, odds ratio effect size*;

+ *F-K index was transformed to area under the curve (AUC)*;

* *p < 0.05*;

** *p < 0.01*;

*** *p < 0.001*.

Stroop protocols were scrutinized for suspected malingering (i.e., color naming *T*-scores over 40; color-word and word reading *T*-scores lower than 40; color and word *T*-scores <40; higher raw scores in color or color-word than in word reading; higher raw scores in color-word than in color naming). Three protocols were classified by these criteria as suspected malingering (alternative hypothesis: low intelligence), a trivial contingency (< 0.05).

### Psychological Adjustment

The results (see Table 2) of the mean comparison between the CPOs with the mean of the normative population (*T* = 50) as test value revealed significantly higher values on all the basic clinical scales. Clinically, CPOs had (*r*) 23% more symptoms of hysteria than the normative population, 37% more depressive symptoms, 44% more hypochondriac symptoms, 68% more psychopathic deviation symptoms, 46% more paranoid symptoms, 26% more psychasthenic symptoms, 24% more symptoms of schizophrenia, 17% more symptoms of hypomania, and 13% more symptoms of social introversion. The magnitude of the effect (see PS_ES_ in Table 2), that is, harm in mental health markers, was above 26.6% of all possible in hypochondria, 43.2% in depression, 51.6% in hysteria, 80.2% in psychopathic deviation, 53.4% in paranoia, 30.4% in psychasthenia, 27.4% in schizophrenia, 19.8% in hypomania, and 14.2% in social introversion.

**Table 2:** One-sample *t*-test for the comparison of CPOs with the mean of the normative population as test value (*T* = 50) in the basic clinical scales.

**Scale**	***t***	***M***	***d***	**PS_**ES**_**	***r***	**AUC**	**PIS(95% CI)**
Hypochondriasis	4.17^***^	56.37	0.48	0.266	0.23	0.633	0.316(0.211, 421)
Depression	6.95^***^	59.90	0.80	0.432	0.37	0.714	0.212(0.119, 304)
Hysteria	8.57^***^	61.25	0.99	0.516	0.44	0.758	0.161(0.078, 0.244)
Psychopathic deviation	15.86^***^	67.81	1.83	0.802	0.68	0.902	0.034(−0.001, 0.075)
Paranoia	8.92^***^	60.95	1.03	0.534	0.46	0.767	0.152(0.071, 0.233)
Psychasthenia	4.75^***^	57.02	0.55	0.304	0.26	0.651	0.291(0.188, 0.394)
Schizophrenia	4.34^***^	56.31	0.50	0.274	0.24	0.638	0.309(0.204, 0.414)
Hypomania	3.01^**^	52.99	0.35	0.198	0.17	0.598	0.363(0.254, 0.472)
Social introversion	2.27^*^	52.90	0.26	0.142	0.13	0.573	0.397(0.286, 0.508)

*df(74). M, mean of the CPO group; d, Cohen's d; PS_ES_, probability of superiority of the effect size; r, incremental in clinical symptoms; AUC, area under the curve; PIS(95% CI), probability of an inferiority score (95% confidence interval)*;

* *p < 0.05*;

** *p < 0.01*,

*** *p < 0.001*.

Moreover, the probability of CPOs having more (AUC) hypochondriac symptoms than the normative population was 63.3, 71.4% more depression, 75.8% more hysteria, 90.2% more psychopathy, 76.7% more paranoia, 65.1% more psychasthenia, 63.8% more schizoid, 59.8% more hypomania, and 57.3% more social introversion.

Epidemiologically (see Table 3), the percentage of clinical deterioration (*T* ≥ 66.45 i.e., percentile 95) in hypochondria (28.0%), depression (29.3%), hysteria (29.3%), psychopathic deviation (60%), paranoia (30.7%), psychasthenia (22.7%), and schizophrenia (25.3%) was significantly higher (*Z*_1_ in Table 3) than expected (0.05 in normative sample). Thus, the rate of caseness in hypochondria was 5.62 times higher than expected (*OR*_1_ in Table 3), 5.86 times higher in depression, 5.86 times higher in hysteria, 12.00 times higher in psychopathy, 6.14 times higher in paranoia, 4.54 times higher in psychasthenia, and 5.06 times higher in schizophrenia. The magnitude of the effect indicated higher rates of caseness than expected (0.05 in normative sample) of 82.1, 82.9, 82.9, 91.7, 83.7, 77.9, and 80.2%, in hypochondria, depression, hysteria, psychopathy, paranoia, psychasthenia, and schizophrenia, respectively. Moreover, the proportion of clinical and moderate deterioration (*T* ≥ 62.8, i.e., percentile ≥ 90) was significantly higher than expected (0.10 in the normative sample) in the same clinical dimensions and in social introversion (*Z*_2_ in Table 3). Succinctly, CPOs experienced 3.07 times more clinical or moderate deterioration in hypochondria (*OR*_2_ in Table 3) than the general population, 4.00 times more depression, 5.20 times more hysteria, 6.13 times more psychopathic deviation, 4.00 times more paranoia, 3.47 times more psychasthenia, 3.07 times more schizophrenia, and 2.40 times more social introversion. This implied an increase in the rate of cases (EII_2_ in Table 3) over the baseline (0.10 in normative sample) of 67.4% for hypochondriasis, 75.0% for depression, 80.8% for hysteria, 83.7% for psychopathic deviation, 75.0% for paranoia, 71.2% for psychasthenia, 67.4% for schizophrenia, and 58.3% for social introversion. Nevertheless, the probability of CPOs being asymptomatic (less symptoms than the mean of the normative sample; see PIS in Table 2) was 31.6% for hypochondriasis, 21.2% for depression, 16.1% for hysteria, 3.4% for psychopathic deviation, 15.2% for paranoia, 29.1% for psychasthenia, 30.9% for schizophrenia, 36.3% for hypomania, and 39.7% for social introversion. These rates were significant, with the exception of psychopathic deviation with an asymptomatic rate of zero (the confidence interval comprises 0).

**Table 3:** Caseness in the MMPI-A basic clinical scales among CPOs.

**Scale**	***f*[*p*(95% CI)]_**1**_**	***Z*_**1**_**	***OR*_**1**_**	**EII_**1**_**	***f*(*p*)_**2**_**	***OR*_**2**_**	***Z*_**2**_**	**EII_**2**_**
Hypochondriasis	21[0.280(0.178, 0.382)]	9.14^***^	5.62	0.821	23(0.307)	3.07	5.98^***^	0.674
Depression	22[0.293(0.190, 396)]	9.54^***^	5.86	0.829	30(0.400)	4.00	8.66^***^	0.750
Hysteria	22[0.293(0.190, 396)]	9.54^***^	5.86	0.829	39(0.520)	5.20	12.12^***^	0.808
Psychopathic deviation	45[0.600(0.489, 0.711)]	21.85^***^	12.00	0.917	46(0.613)	6.13	14.81^***^	0.837
Paranoia	23[0.307(0.203, 0.411)]	10.33^***^	6.14	0.837	30(0.400)	4.00	8.66^***^	0.750
Psychasthenia	17[0.227(0.132, 0.322)]	7.03^***^	4.54	0.779	26(0.347)	3.47	7.13^***^	0.712
Schizophrenia	19[0.253(0.155, 0.351)]	7.94^***^	5.06	0.802	23(0.307)	3.07	5.98^***^	0.674
Hypomania	5[0.067(0.010, 0.124)]	0.79	1.34	0.254	8(0.107)	1.07	0.20	0.065
Social introversion	6[0.080(0.019, 0.141)]	1.19	1.60	0.375	18(0.240)	2.40	4.04^***^	0.583

*df(74). f[p(95% CI)]_1_, frequency of clinical deterioration[observed probability (95% confidence interval)]; Z_1_, zeta score for the difference between the observed proportion of clinical deterioration among CPOs and a constant (0.05, predicted probability of clinical deterioration in the normative sample); OR_1_, odds ratio for the clinical deterioration; EII_1_, effect incremental index for clinical deterioration; f(p)_2_, frequency of clinical and moderate deterioration(observed probability); OR_2_, odds ratio for clinical and moderate deterioration; Z_2_, zeta score for the difference between the observed proportion of clinical and moderate deterioration among CPOs and a constant (0.10, predicted probability of clinical and moderate deterioration in the normative sample); EII_2_, effect incremental index for clinical and moderate deterioration*;

*** *p < 0.001*.

Furthermore, the results of the comparison of the means of CPOs with the test value of clinical cases (*T* = 66.45) showed this population (see Table 4) was characterized by psychopathic deviation (the mean is over the criterion for clinical deterioration classification, 66.45, as the lower limit of the confidence interval is above this cut-off score); for hysteria and paranoia, CPOs were in the region of moderate deterioration (the confidence interval of the mean for CPOs was 62.8, the criterion for the classification of moderate deterioration). However, hypochondriasis, depression, psychasthenia, schizophrenia, hypomania, and social introversion were within the limits of normality (the upper limit of the confidence intervals is under 62.8).

**Table 4:** One-sample *t*-test for the mean comparison of CPOs with the cut-off score for caseness as test value (*T* = 66.45) in the basic clinical scales.

**Scale**	***t***	***M*(95% CI)**	***d***
Hypochondriasis	−6.61^***^	56.37(53.37, 59.37)	−0.76
Depression	−4.59^***^	59.90(57.10, 62.70)	−0.53
Hysteria	−3.97^***^	61.25(58.68, 63.52)	−0.46
Psychopathic deviation	1.21	67.81(65.61, 70.01)	0.04
Paranoia	−4–48^***^	60.95(58.54, 63.36)	−0.52
Psychasthenia	−6.38^***^	57.02(54.12, 59.92)	0.0.74
Schizophrenia	−6.98^***^	56.31(53.47, 59.15)	−0.81
Hypomania	−13.56^***^	52.99(51.05, 54.93)	−1.56
Social introversion	−10.62^***^	52.90(50.39, 55.41)	−1.23

*df(74). M(95% CI), mean of the CPO group(95% confidence interval); d, Cohen's d*;

*** *p <0.001*.

The comparison of the mean of the CPOs with the weighted-by-sampling-size mean—from 21 to 23 samples and none of CPOs—of the juvenile justice samples (Baum et al., 2009) showed significantly more hypochondriac, depressive, hysteric, psychopathic, paranoid, and psychasthenic clinical symptoms among CPOs (see Table 5). In terms of the increase in clinical symptoms, CPOs reported 20.0% more hypochondriac, 24.7% more depressive, 46.1% more hysteric, 42.2% more psychopathic deviation, 16.3% more paranoid, and 14.8% more psychasthenic symptoms than juvenile justice samples. Conversely, CPOs reported less hypomanic clinical symptoms than justice juveniles, specifically 15.3% less hypomanic symptoms.

**Table 5:** One-sample *t*-test for the mean comparison of CPOs with the mean of juvenile justice samples as test value in the basic clinical scales.

**Scale**	***t***	***M*_**CPO**_**	***M*_**JJS**_**	***d***	***r***
Hypochondriasis	3.54^***^	56.37	50.97	0.41	0.200
Depression	4.46^***^	59.90	53.55	0.51	0.247
Hysteria	8.99^***^	61.25	49.45	1.04	0.461
Psychopathic deviation	8.03^***^	67.81	58.79	0.93	0.422
Paranoia	2.89^**^	60.95	57.40	0.33	0.163
Psychasthenia	2.61^*^	57.02	53.16	0.30	0.148
Schizophrenia	0.97	56.31	54.90	0.11	0.055
Hypomania	−2.71^**^	52.99	55.68	−0.31	−0.153
Social introversion	1.66	52.90	50.78	0.19	0.095

*df(74). M_CPO_, mean of the CPO group; M_JJS_, mean of the juvenile justice samples; d, Cohen's d; r, incremental in clinical symptoms*;

* *p < 0.05*;

** *p < 0.01*;

*** *p < 0.001*.

### Executive Functioning

The results showed CPOs had impairment in word reading, color naming, color-word, and interference (see Table 6). The magnitude of the impairment (*r*) was estimated as 62.0% in word reading, 47.9% in color naming, 45.8% in color-word, and 11.9% in interference score.

**Table 6:** One-sample *t*-test for the mean comparison between CPOs and the cut-off score for impairment (*T* = 40) as test value in the Stroop tasks.

**Variable**	***t***	***M***	***d***	***r***
Word reading	−13.76^***^	39.78	−1.08	−0.620
Color naming	−9.08^***^	39.25	−1.07	−0.479
Color-Word	−8.86^***^	40.29	−0.97	−0.458
Interference score	−2.06^*^	48.18	−0.19	−0.119

*df(74). M, mean of the CPOs; d, Cohen's d; r, incremental in clinical symptoms*;

* *p < 0.05*;

*** *p < 0.001*.

The study of cases (*T* ≤ 40; see Table 7) revealed a significantly higher than expected prevalence in the general population of caseness (0.1587) in word reading, color naming, and color-word, but not in resistance to interference. The magnitude of the deterioration of a medium effect size (*OR* > 2.47) indicated an increase (EII) in the observed proportion of impairment over the baseline of 65% in word reading, 71% in color naming, and 70.2% in color-word.

**Table 7:** Caseness in the Stroop tasks among CPOs.

**Variable**	***f*(*p*)**	***Z***	***OR***	**EII**
Word reading	34(0.453)	6.98^***^	2.85	0.650
Color naming	41(0.547)	9.20^***^	3.45	0.710
Color-word	40(0.533)	8.87^***^	3.36	0.702
Interference score	10(0.133)	−0.61	0.83	−0.162

*Classification criterion as impairment: T < 40; f(p), frequency of impairment(proportion); Z, zeta score for the difference of the registered proportion of impairment among CPOs with a constant (0.1587, expected probability for a T score of 40); OR, odds ratio; EII, effect incremental index*;

*** *p < 0.001*.

## Discussion

The generalization of the results is subject to limitations that should be borne in mind. First, the sensitivity of the design for the case study was not optimum (α/β ≠ 1) and biased against finding significant ratios of clinical deteriorate caseness (1 – β < 0.80). Thus, the significance of clinical deterioration caseness may be higher than found, so special attention should be paid to CIs. Second, although general and intended malingering and defensive responding was ruled out, the combination of both should be suspected in the case study as indicators of malingering and defensiveness may be insufficient by themselves (Osuna et al., 2015). Third, the Stroop tasks involve relatively simple tasks in experimental settings. The direct generalization of the results from experimental settings to real context is problematic. Thus, subjects who do not display impairment on Stroop tasks may have difficulties in everyday tasks requiring executive control (Fariña et al., 1994).

The CPOs cooperated with the psychological evaluation (Cannot Say Scale < 10) and exhibited a consistent response pattern (TRIN and VRIN rs < 14). Nevertheless, a significant number of CPOs changed their response style throughout the test. In all cases (*n* = 15), *F1* was greater than *F2* (> +16.45); as *F1* and *F2* inform of suspected malingering, this index indicated the suspicion of malingering was greater on the basic clinical scales (*F1*) than on the content and complementary scales (*F2*). However, as it is easier to malinger on the content and complementary scales than on the basic clinical scales (Greene, 2011), the change in response style cannot be attributed to intentional manipulation, but rather to response characteristics of the people under evaluation (alternative hypothesis). Succinctly, an intentional change in response style was ruled out.

Likewise, CPOs' item responses were accurate, i.e., were not biased either in terms of malingering with the presence of psychopathology or socially undesirable characteristics or in defensiveness. Moreover, the clinical profiles ruled out two key malingering strategies (Vilariño et al., 2013; Rogers, 2018): indiscriminate grouping of symptoms (no reported harm in all of the disorders; in fact, hypochondriasis, depression, psychasthenia, schizophrenia, hypomania, and social introversion report normality) and symptom severity (participants reported no severe disorder, *T* < 90). In short, the systematic tendency to malinger was ruled out in CPOs, i.e., a diagnostic criterion for CD and a differential diagnosis for PTSD in forensic setting (American Psychiatric Association, 2000, 2013), a diagnosis with significant rates [61.7% (95% CI 55.4–67.9%) of CD and 8.6% (95% CI 6.4–10.7%) of PTSD] among juvenile offenders (Beaudry et al., 2021). Likewise, systematic defensiveness (defensiveness may consist in denial of symptoms and/or the adoption of desirable characteristics—social desirability—to mask an unfavorable image; Arce et al., 2015; Rogers, 2018) was ruled out in CPOs: *L, K*, and the *F-K* index ruled out the suspicion of defensiveness.

Nonetheless, a significant number of CPOs were classified by the *F* scale (66 items) and by the *F1* (33 items) and *F2* (33 items) scales that are subdivisions of the *F* scale as suspected malingering, but not by the *K* scale and the *F-K* index. As only one indicator classified malingering, the *F* scale (*F1* and *F2* were part of *F* and would lead to a duplicity of measures if counted independently), this criterion was insufficient for suspecting malingering (Graham, 2011; Greene, 2011; Fariña et al., 2014; Arce, 2018), meaning other alternative hypotheses had to be considered: inconsistent response pattern (previously ruled out) and severe psychopathology (Greene, 2011; Arce, 2018). As the strategies of severity and the indiscriminate grouping of symptoms (discriminant validity) were ruled out, and the reported clinical profiles were consistent with those registered in juvenile justice samples (convergent validity; Baum et al., 2009), the alternative hypothesis to malingering, severe psychopathology, was accepted (Arce, 2018). As for defensiveness, the *L* scale classified a significant number of CPOs as suspected of social desirability response bias (denial of personal faults; Rogers, 2018). Once again, as only one indicator of defensiveness (*K* and *F-K* do not classify significantly defensiveness) was significant, it was insufficient for classifying CPOs as defensiveness biased responses (Arce et al., 2015).

Clinically, CPOs experience more symptoms on the basic clinical dimensions than the normative population, but only report clinical deterioration in psychopathic deviation (unreliable, egocentric, and irresponsible; unable to learn from experience and to plan ahead; problems with family members and authority; anger toward others; and problematic interpersonal relations in large interactions and under stress); and moderate deterioration in hysteria (naive, self-centered, denying any problem, exhibitionist, extroverted, and superficial) and paranoia (highly sensitive to criticism and to personalize the actions of others toward themselves). Furthermore, CPOs report more clinical maladjustment than other juvenile justice samples, which is characterized by psychological problems (Baum et al., 2009; Marcos et al., 2020; Beaudry et al., 2021). Hence, the population of CPOs are a clinical population experiencing more deterioration than other samples of juvenile justice. Epidemiologically, the observed prevalence of clinical and moderate deteriorated caseness was extremely high (incremental rate above baseline > 67.4%) in all the clinical dimensions excluding hypomania and social introversion. Thus, clinical and moderate caseness were diverse and comorbid (or multi-comorbid).

The results of the CPO population in Stroop tasks suggest an impairment in word reading, color naming, and color-word tasks, as well as in interference. These results were linked to deficits in working memory (Long and Prat, 2002); dysfunctions in selective attention (Fanti et al., 2016); poor cognitive flexibility or dysfunctions in cognitive inhibition (Lee and Orsillo, 2014); and poor abilities of goal formation and planning, carrying out goal-directed plans, and effective performance (Jurado and Rosselli, 2007). That is, poor skill at inhibiting responses linked to stimuli requiring the suppression of automatic responses (Herrero et al., 2019). Additionally, the case study has shown a significantly high rate of caseness among CPOs. These neuropsychological impairments appear to be linked to the onset, maintenance, and abandonment of antisocial behavior (Séguin, 2009).

Finally, the comorbidity of a clinical disorder with deficits in executive functioning is a characteristic of the CPO population. As the clinical intervention and training of executive functions are effective, and cognitive bias may play a role in the maintenance of psychopathology (Mogg and Bradley, 2005; Soriano et al., 2020), a key objective of interventions with CPOs should be the clinical treatment and the training of cognitive functions necessary for self-regulation and the regulation of socially appropriate behavior.

Future research should be focused to ascertain if impairments in EFs are characteristic of juvenile offenders (or offenders in general) or specific to CPOs and if the deficits in psychological adjustment and EFs are combined with deficits in social and cognitive competence.

## Data Availability Statement

All relevant data are within the article and its supporting information files.

## Ethics Statement

Ethical review and approval was not required for the study on human participants in accordance with the local legislation and institutional requirements. Written informed consent from the participants' legal guardian/next of kin was not required to participate in this study in accordance with the national legislation and the institutional requirements.

## Author Contributions

All authors listed have made a substantial, direct and intellectual contribution to the work, and approved it for publication.

## Conflict of Interest

The authors declare that the research was conducted in the absence of any commercial or financial relationships that could be construed as a potential conflict of interest.
